# Patient perspectives on Peyronie’s disease: results of poststudy interviews from a phase 2 trial of collagenase clostridium histolyticum

**DOI:** 10.1038/s41443-018-0027-5

**Published:** 2018-09-20

**Authors:** J. Kaminetsky, M. Gittelman, G. J. Kaufman, T. M. Smith, G. H. Jordan

**Affiliations:** 1 Manhattan Medical Research, New York, NY USA; 2 21st Century Oncology/UroMedix-Aventura Division, Aventura, FL USA; 3 Auxilium Pharmaceuticals, Inc., Chesterbrook, PA USA; 4 Eastern Virginia Medical School, Norfolk, VA USA

**Keywords:** Quality of life, Translational research

## Abstract

Intralesional injection of collagenase clostridium histolyticum (CCH) improves Peyronie’s disease (PD) symptoms; however, patient perspectives regarding PD and CCH treatment have not been fully elucidated. This cross-sectional qualitative study included heterosexual men with PD who received ≥1 injection of study medication and had ≥1 posttreatment Peyronie’s Disease Questionnaire (PDQ) assessment during a prior phase 2b clinical trial. These patients were “responders” if they reported (as part of the Global Assessment of the PDQ) that overall symptoms and effects of PD had at least “improved in a small but important way” after CCH therapy. Among 45 patients interviewed, penile bending or curvature was the most common and bothersome PD symptom reported (by 97.8% and 48.9% of patients, respectively). Patients indicated that multiple alterations were necessary in their sex lives because of penile symptoms and specified that these changes impacted their emotional health and partner relationship. Treatment with CCH improved PD symptoms (44.4%), frequency of or ability to have vaginal intercourse (22.2%) and partner relationship (22.2%), particularly among responders. Given that physical, psychologic and sexual function are impacted by PD, clinical trials that evaluate treatments for PD should include patient-reported outcome measures (e.g., the PDQ) to assess overall well-being after treatment.

## Introduction

Peyronie’s disease (PD) is characterized by reduced extensibility of the tunica albuginea as the result of aberrant wound healing after trauma [1]. In normal circumstances, the exposure of fibrin as the result of trauma initiates an inflammatory cascade that causes the differentiation of fibroblasts into collagen-producing myofibroblasts [1]. As the healing process progresses, myofibroblasts undergo apoptosis, resulting in reduced collagen production [1]. In PD, myofibroblasts remain active and lead to the accumulation of collagen and the development of fibrotic penile plaques [1, 2].

Many urologists consider PD a rare condition [3] and think that PD symptoms remit spontaneously (i.e., do not require treatment) [3]. However, the literature suggests that 3.2–11.8% of men experience PD, depending on the country, with a higher prevalence among older individuals [4–6], and over the course of about 1 year, only 12% of patients have shown improvement in PD symptoms without treatment [7]. In fact, many (48%) patients have worsening of symptoms [7] and warrant treatment. In addition, although PD is not a life-threatening condition, it may have a substantial impact on patient physical [7], psychologic [8–11], sexual, and relationship health [7, 8, 11, 12]. Symptoms such as penile shortening and deformity (e.g., curvature) are prevalent in PD [7] and may disrupt the ability to perform sexually [10, 12] and negatively impact body image [10] and personal relationships [12].

Collagenase clostridium histolyticum (CCH) has been approved by the US Food and Drug Administration (FDA) for the treatment of adult men with PD with a palpable plaque and curvature deformity of ≥30° at the start of therapy [13]. In phase 2b [14] and phase 3 trials [15], intralesional CCH injections significantly improved penile curvature and the degree of symptom bother in patients with PD. The Peyronie’s Disease Questionnaire (PDQ) and the Global Assessment of the PDQ (GAPDQ) were created during the clinical development program of CCH for PD to assess the psychologic and social impacts of treatment (i.e., the PDQ) and establish overall treatment efficacy (i.e., GAPDQ). As part of the phase 2b trial of CCH for the management of PD, a cognitive debriefing interview that consisted of individual patient interviews was performed to provide qualitative research on patients’ experiences with PD and its treatment.

## Materials and methods

This was a cross-sectional, qualitative study conducted with men who had participated in a phase 2b trial of CCH for treatment of PD. All 12 US sites from the phase 2b trial were invited to participate, and six elected to continue in the current study. Details on the study design and patient population for the randomized, double-blind phase 2b study have been published previously [14]. Briefly, men ≥18 years of age with PD who were in a stable heterosexual relationship for ≥3 months (*n* = 147) were included and stratified by degree of penile curvature (30°–60° or >60°). Patients were excluded if they had penile curvature <30° or >90°, ventral penile curvature of any etiology, had calcified plaques or any plaques that would have interfered with injections, displayed isolated hourglass deformity of the penis without curvature, reported severe pain during penile palpation, or did not get a full erection after prostaglandin E1 treatment.

In the phase 2b study, patients were randomly assigned 3:1 to receive CCH 0.58 mg or placebo and then subsequently randomized in a 1:1 ratio to either receive or not receive penile plaque modeling. Patients could receive up to three cycles of CCH treatment (two injections separated by 24–72 h), with each cycle separated by 6 weeks. Efficacy measurements included the degree of penile curvature and patient-reported PDQ and GAPDQ scores, all three of which were evaluated at weeks 18, 24, and 36 after the last injection. Scores for the fifth item of the GAPDQ (rating of the overall symptoms and effects of PD; scored from 3 [much improved] to –3 [much worse]) at week 36 determined responder status (“responder”: score ≥1 [at least “improved in a small but important way” after treatment]; nonresponder: score < –1 [at least “a little worse” after treatment]).

In the current analysis, patients from six US study sites were recruited if they had received at least one treatment cycle of study medication (CCH or placebo) and had at least one PDQ assessment after treatment. Patient interviews were conducted between March 5, 2010, and March 25, 2010, to explore the physical symptoms of PD, the impact that PD-related functional and psychologic disruptions had on the patient, and patient experiences before and after the trial. Each 1-hour, one-on-one interview was conducted by clinical research staff via telephone using a semistructured interview guide that contained queries and prompted patient perceptions of the effects of the study medication. All reports of experiences (i.e., pretrial and posttrial appraisals) were based on patient recall because all interviews were conducted after the trial had ended. After the telephone interviews, transcripts were coded by staff who were blinded to treatment group. These coded records (interview notes and digital records) were then examined to determine relevant themes during each time period (i.e., before and after the trial). Pretrial themes included identification of the most bothersome or distressing symptoms, diagnosis and prior treatment and the impact of symptoms (e.g., impact on sexual partner, alterations made to sex life). During the trial, themes identified were changes in symptoms, including the most bothersome or distressing symptoms, and the impact of symptoms. Post-trial themes probed treatment satisfaction, treatment expectations, the status of symptoms and the impact of PD symptoms on daily living. Qualitative interview data were analyzed using ATLAS.ti software version 5.6.3 (ATLAS.ti Scientific Software Development GmbH, Berlin, Germany), employing a content analysis approach. Demographic and clinical data were summarized descriptively. The posttreatment study protocol was approved by Ethical Review Committee, Inc (Independence, MO, USA). All patients provided written informed consent.

## Results

Of the 75 patients who met eligibility criteria, 60 (80.0%) were interested in participating in the posttreatment interview; 45 of these 60 patients (75.0%) consented and completed the interview. Patients were not interviewed if they withdrew consent after speaking further with study staff (*n* = 5) or provided consent after target enrollment (i.e., determined by saturation of concepts) had been reached (*n* = 10). Most of the 45 patients who completed the interviews identified as white, did not have a history of penile pain, did not have a history of erectile dysfunction and received CCH during the clinical trial (Table 1).

**Table 1:** Demographic and baseline characteristics

Characteristics	Patients (*n* = 45)
Mean age, year (s.d.)	57.3 (7.9)
Race, *n* (%)	
White	42 (93.3)
Black/African American	3 (6.7)
Treatment received during the clinical study, *n* (%)	
CCH	33 (73.3)
Placebo	12 (26.7)
History of ED, *n* (%)	22 (48.9)
Mean time since PD diagnosis, year (s.d.)	2.8 (2.5)
Duration of penile pain, *n* (%)	
No pain	34 (75.6)
<3 months	0
3–6 months	1 (2.2)
6–9 months	1 (2.2)
>9 months	9 (20.0)
Time between first notice of penile changes and study entry	
6 months–1 year	2 (4.4)
1–2 years	13 (28.9)
2–5 years	17 (37.8)
>5 years	12 (26.7)
Not reported	1 (2.2)
Other medications used for PD, *n* (%)	
None	17 (37.8)
Oral medications/vitamins	23 (51.1)
Creams/topical ointments	10 (22.2)
Stretching/vacuum devices	6 (13.3)
Injections	5 (11.1)
Treatment during phase 2b trial, *n* (%)	
CCH group	*n* = 33
Week 36 GAPDQ responder^a^	20 (60.6)
Week 36 GAPDQ nonresponder	11 (33.3)
Not evaluable^b^	2 (6.1)
Placebo group	*n* = 12
Week 36 GAPDQ responder^a^	4 (33.3)
Week 36 GAPDQ nonresponder	6 (50.0)
Not evaluable^b^	2 (16.7)

*CCH* collagenase clostridium histolyticum, *ED* erectile dysfunction, *GAPDQ* Global Assessment of the Peyronie’s Disease Questionnaire, *PD* Peyronie’s disease, *s.d.* standard deviation

^a^GAPDQ responders had GAPDQ question 5 score ≥ 1 at posttreatment week 36

^b^No GAPDQ assessment at posttreatment week 36

### Patient perceptions before treatment

When asked about the changes noticed in their penises during onset of PD, 44 of the 45 patients (97.8%) reported bending or curving of the penis. The most frequent penile changes were penile shortening (*n* = 28; 62.2%), changes in penile shape (*n* = 21; 46.7%), presence of penile plaque or lesions (*n* = 20; 44.4%) and pain in the erect penis (*n* = 21; 46.7%). The most distressing or bothersome symptoms reported by patients (*n* = 45) during onset of PD included bend or curvature of the penis (*n* = 22; 48.9%), changes in vaginal intercourse (*n* = 20; 44.4%), appearance of the penis (*n* = 17; 37.8%) and frequency of vaginal intercourse (*n* = 16; 35.6%). More than half of patients (*n* = 24; 53.3%) indicated that the distressing or bothersome symptoms negatively affected their psychologic well-being.

**Fig. 1:**
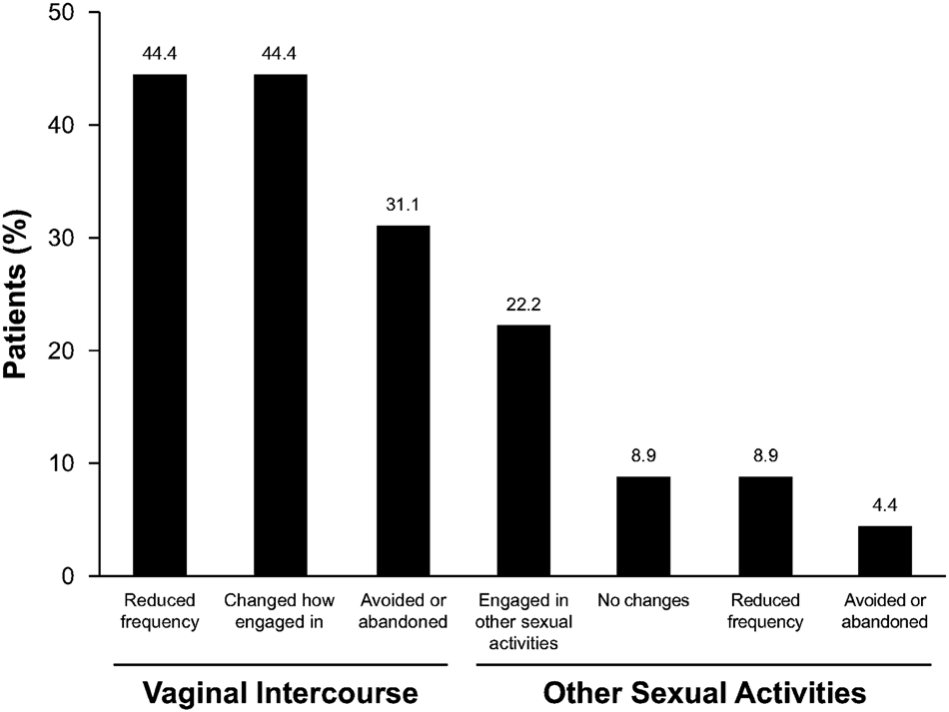
Patient-reported alterations made to their sex lives because of Peyronie’s disease (*n* = 45). Responses were given in response to the question “What changes did you make to your sex life as a result of these [physical] changes to your penis?”

**Fig. 2:**
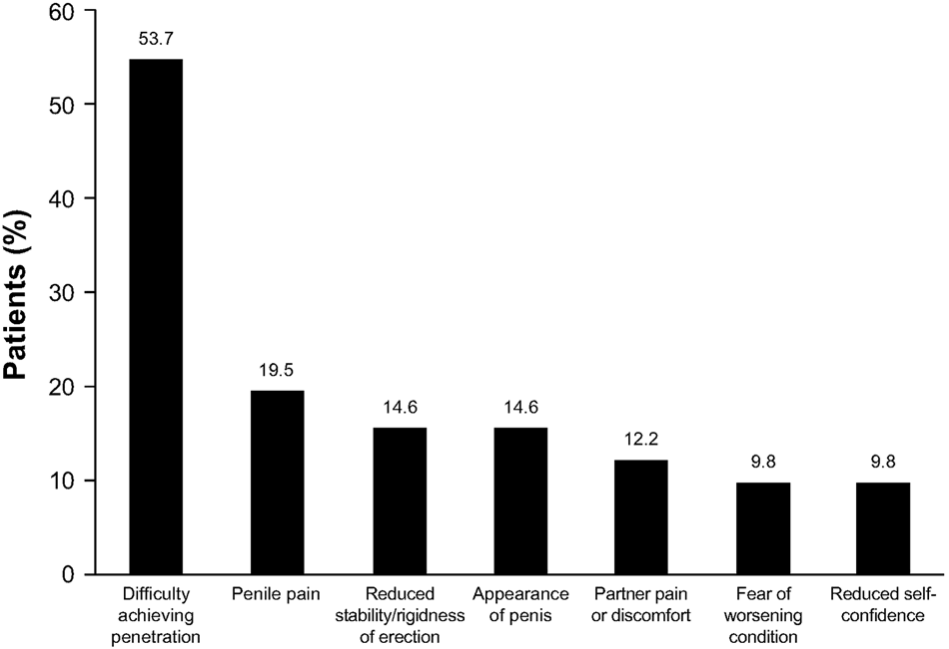
Patient-reported reasons for changes in their sex lives (*n* = 41). Responses were prompted by the question “Why did you make these changes [in your sex life]?”

**Fig. 3:**
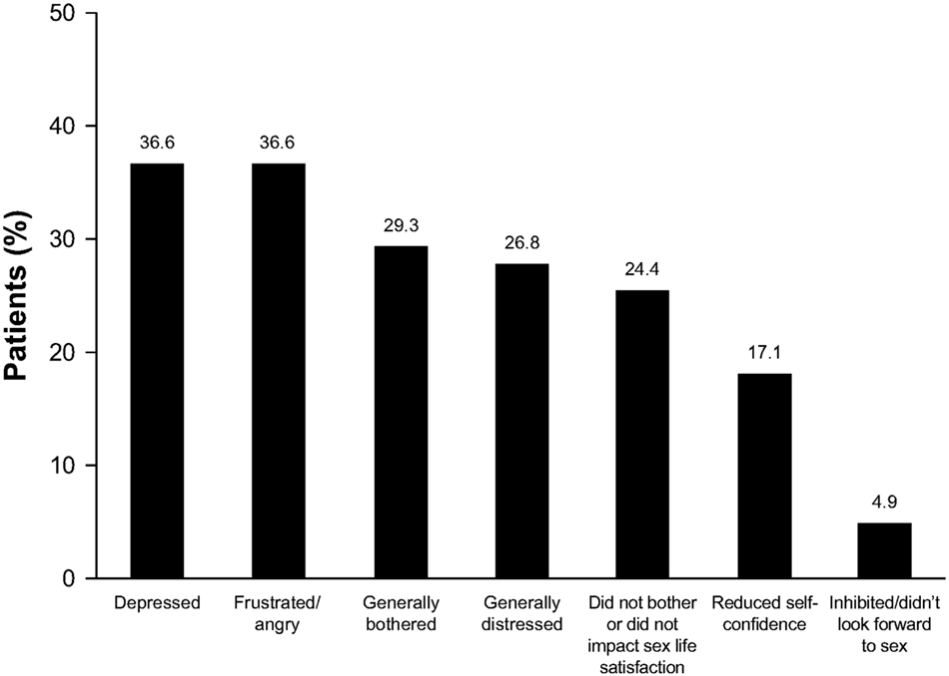
Patient feelings about alterations in their sexual activity because of Peyronie’s disease (*n* = 41). Feelings were given in response to the question “How did you feel about changes to your sex life?” Each patient may have reported more than one feeling

Patients reported multiple changes in their sex lives related to their penile symptoms that were associated with PD onset (*n* = 45; Fig. 1). The most common changes included a reduction in the frequency of vaginal intercourse and alterations in how patients engaged in vaginal intercourse (*n* = 20; 44.4% for each). Among patients who responded to the question about why they made changes to their sex lives (*n* = 41), difficulty achieving penetration during vaginal intercourse was the most commonly reported reason (Fig. 2). Of the 41 patients who responded to the question of how they felt about changes they had made to their sex lives, most were depressed or frustrated/angry about the alterations (Fig. 3). Only eight (19.5%) patients said that the changes did not bother them, and only two (4.9%) indicated that the changes did not impact their satisfaction with their sex lives. Most patients (*n* = 25; 61.0%) reported that their sexual partner was supportive of or understanding about the alterations in their sex life because of their situation. However, some of the 41 patients thought that their partner experienced depression (*n* = 5; 12.2%), frustration (*n* = 3; 7.3%), disappointment (*n* = 2; 4.9%), distress (*n* = 2; 4.9%), bother (*n* = 2; 4.9%) and/or reduced satisfaction with sex (*n* = 2; 4.9%).

In some instances (three of the 45 interviewed patients; 6.7%), PD was thought to have contributed to the end of the relationship with their partner, although the majority of patients (*n* = 27; 60.0%) expressed that PD did not impact their relationship with their sexual partner. Regarding expectations before initiating treatment, 24.4% of the 45 patients were hoping for their penis to return to its normal appearance, and another 24.4% were hoping for an improvement in penile curvature or shape.

### Perceptions during and after clinical trial participation

Almost half (*n* = 20; 44.4%) of the 45 patients reported improvement in at least one of their PD symptoms during or after the trial. Of these 20 patients, 14 (70.0%) were classified as responders. The overall population of 45 patients most commonly reported improvement in bend or curvature of the penis (51.1%), but improved ability to have vaginal intercourse (22.2%) and improved size or firmness of the plaque lesions (20.0%) were also frequently reported, mostly among responders (79–100% of responders). Approximately half of the 45 patients (*n* = 23; 51.1%) indicated a change in their most bothersome symptoms after the trial. Of these 23 patients, 43.5% (*n* = 10) reported improvement in the curvature or shape of their penis (nine out of those ten were responders) and 30.4% (*n* = 7) reported improvement in the frequency of or ability to have vaginal intercourse, or improvement in both of these areas. Many (*n* = 19; 42.2%) of the 45 patients reported that their self-confidence to engage in vaginal intercourse improved after the trial. Of these 19 patients, 12 were responders.

**Fig. 4:**
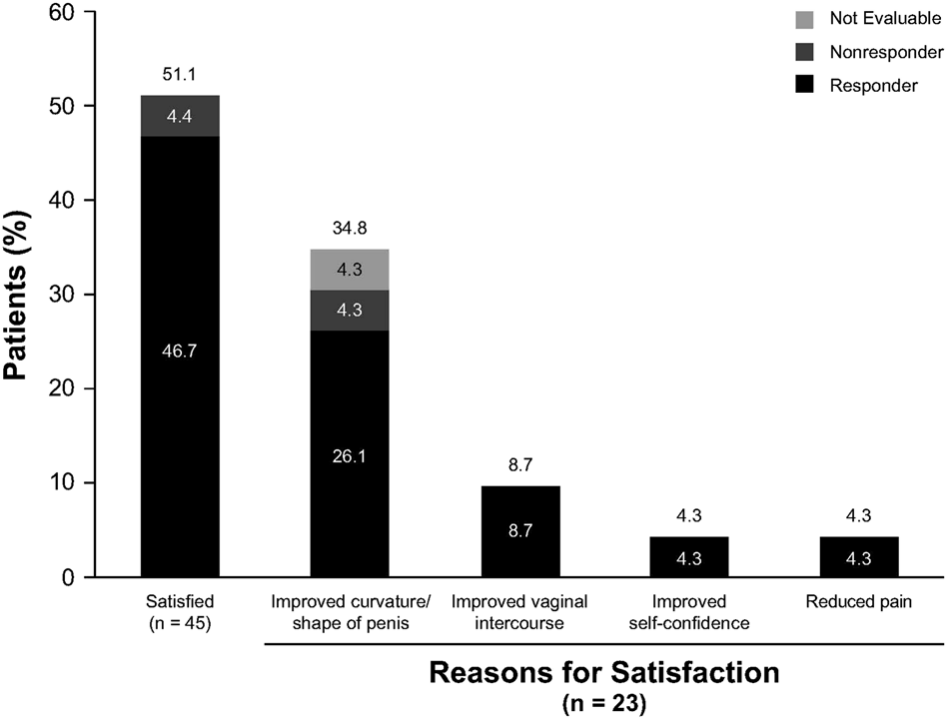
Percentage of patients reporting satisfaction and the reason for satisfaction with treatment. Responders were patients who scored ≥1 (at least “improved in a small but important way” after treatment) in response to the fifth question of the Global Assessment of the Peyronie’s Disease Questionnaire (GADPQ; rates overall symptoms and effects of Peyronie’s disease on a scale from 3 [much improved] to –3 [much worse]). Nonresponders were patients with GADPQ scores < –1 (at least “a little worse” after treatment) on the same question

Almost half (48.9%) of the 45 patients categorized their treatment as successful, and as shown in Fig. 4, 51.1% (*n* = 23) were satisfied or pleased with their results. Satisfaction was more common among responders than nonresponders. The main reason for satisfaction cited by the 23 patients was improvement in the curvature or shape of their penis (Fig. 4). The majority of patients (55.6%; *n* = 25) were also at least somewhat satisfied with their sex lives after completing the clinical trial; 60.0% of these patients were responders. In addition, 22.2% (*n* = 10) noticed a positive change in the relationship with their partners. This positive change was reported more often by responders (*n* = 7 of ten patients). Only 46.7% of the 45 patients indicated that expectations for their treatment had been met or somewhat met. Of the 45 patients, 42.2% reported that their sexual partners were satisfied or somewhat satisfied with the treatment the patient had received. Five of the 45 patients (11.1%) indicated partner satisfaction was related to improved sexual intercourse.

## Discussion

The results of this series of cognitive debriefing interviews provide additional understanding of the physical, psychologic and social obstacles patients with PD encounter, and the ways in which effective treatment eases those struggles. These insights may allow health care providers to more effectively communicate with patients about PD symptoms and set reasonable posttreatment expectations.

In this study, patients indicated that their most bothersome or distressing symptoms before treatment were penis appearance (e.g., bend or curvature of the penis), changes in vaginal intercourse and frequency of vaginal intercourse. Penile bending or curvature may make it difficult or limiting for patients to engage in sexual intercourse and might contribute to reductions in sexual activity [10, 12]. Indeed, reduced frequency of vaginal intercourse was one of the most commonly reported PD-related alterations that patients made to their sex lives. Patients also frequently reported being depressed, frustrated, or angry because of alterations to their sex lives.

The combination of negative emotions and reduced frequency of sex may substantially impact the emotional and sexual health of patients and their partners. For example, relationship difficulties in men with PD have been reported frequently [11, 12, 16], and in the current study, one-fifth of patients said that PD negatively impacted their relationships with their partners. Although the factors underlying these relationship difficulties are likely diverse, emotional difficulties and the ability to have intercourse both have been shown to predict relationship problems [11]. Furthermore, catastrophizing, increased bother or distress and reduced sexual frequency have been reported to negatively impact sexual satisfaction in men with sexual difficulties [16–18]. However, female partners of men with sexual dysfunction may not be as impacted by these symptoms as was once thought. For instance, in the International Survey of Relationships study, female partners of men with sexual problems reported that their relationship happiness was not substantially impacted by the sexual difficulties of their male partners, although their partners reported reduced sexual satisfaction [17]. Furthermore, men with sexual problems tended to overestimate the impact of their sexual difficulties on their female partners [17]. Nonetheless, pharmacologic therapies such as CCH that improve the physical manifestations of the disease (e.g., penile deficiencies [15]) could also potentially reduce distress, improve the quality of life of patients and improve their relationships.

Most patients in the current study indicated that their bothersome symptoms had improved after CCH administration, with many patients reporting improvements in penile curvature or shape, the frequency of or ability to have vaginal intercourse and increased self-confidence to engage in vaginal intercourse. Given that greater frequency of intercourse tends to improve relationship status [18], appropriate management of PD with CCH may impact physical as well as sexual and relationship satisfaction. The results of previous CCH studies, including the phase 3 clinical trials, have shown improvements in both physical and bother domain scores with CCH treatment [14, 15, 19, 20]. In the current study, this finding is supported by the rating of penile curvature/shape and improved vaginal intercourse as the most commonly reported reasons for satisfaction with CCH treatment. However, only a moderate percentage of patients reported that CCH treatment had met their expectations. This may be attributable to a disparity between high patient expectations (e.g., complete reversal of the condition) and actual treatment efficacy, and emphasizes the importance of setting realistic patient expectations in the clinical setting.

This study had a number of limitations. Patient perception of symptoms and attitudes toward their disease were collected using a nonvalidated questionnaire, and the definition of treatment response was based on a patient-reported outcome assessment (i.e., the GAPDQ). The FDA has become critical of patient-reported outcome assessments during the last several years [21]. However, in general, the results of the GAPDQ assessment of treatment efficacy (i.e., patients classifying themselves as a “responder” or “nonresponder”) used in this study were essentially confirmed by individual interview responses because those reporting symptom improvements during interviews were generally treatment responders.

Other limitations were that the current study examined a limited number of patients in the United States who participated in the phase 2b clinical trial, and that interview responses were based on patient recall after the end of the clinical trial. Despite these limitations, the findings presented herein provide health care providers important insights into the potential challenges and limitations experienced by patients with PD. It would be interesting to prospectively examine the psychologic, sexual, and physical obstacles of a larger, more diverse patient population, especially one including patients who experienced penile pain or had concomitant erectile dysfunction, whose numbers were limited in the current study.

In summary, patients with PD face a number of potentially debilitating physical symptoms. The results of this cognitive debriefing interview series suggest that the impact of PD on psychologic and sexual function is at least as important as its physical impact. Because of this, it is important that clinical trials examining treatments for men with PD include patient-reported outcome measures, such as the PDQ, that evaluate a patient’s overall well-being after treatment. In addition, it is important that health care providers reinforce and manage the treatment expectations of patients and their sexual partners before PD therapy begins.
